# Combined effect of underwater ultrasound and custom-made insoles in rheumatoid arthritis: a randomized controlled trial

**DOI:** 10.1007/s10067-026-08076-0

**Published:** 2026-03-28

**Authors:** Ömer Faruk Özçelep, Ceren Demir, Elife Ceyda Okumuş, Mehmet Canlı, Halil Alkan

**Affiliations:** 1 School of Physical Therapy and Rehabilitation, Kırşehir Ahi Evran University, Kırşehir, Turkey; 2 Department of Rheumatology, Kırşehir Training and Research Hospital, Kırşehir, Turkey; 3 Department of Physiotherapy and Rehabilitation, Muş Alparslan University, Muş, Turkey

**Keywords:** Custom-made insole, Plantar pressure, Rheumatoid arthritis, Ultrasound

## Abstract

**Background:**

To investigate the effects of underwater ultrasound therapy alone and in combination with custom-made insoles in individuals with rheumatoid arthritis (RA).

**Methods:**

This single-blind, randomized controlled trial included 75 patients with mild-to-moderate RA and foot pain, who were randomly allocated into three groups: control group (CG), underwater ultrasound group (UG), and underwater ultrasound plus custom-made foot insole group (IG). Interventions were applied over a 6-week period. Outcome measures included the Health Assessment Questionnaire (HAQ), Manchester–Oxford Foot Questionnaire (MOFQ), Timed Up and Go test (TUG), Berg Balance Scale (BBS), Static Plantar Pressure Distribution (SPPD), Single Heel-Rise Test (SHRT), and ankle circumference (AC). Assessments were performed before and after the intervention period.

**Results:**

Significant time effects were observed for most outcome measures, indicating overall improvements after the intervention period across all groups (*p* < 0.05), except for AC. A significant group × time interaction was found for HAQ (*p* = 0.002, *η*^2^ = 0.209) and selected SPPD parameters (RF, RB, and LB; *p* < 0.05), suggesting differential changes over time between groups. No significant group × time interactions were observed for MOFQ, TUG, BBS, SHRT, or AC, indicating comparable clinical improvements among groups. In contrast, SPPD demonstrated significant between-group differences, particularly in the group receiving custom-made foot insoles (*p* < 0.05).

**Conclusion:**

Underwater ultrasound therapy was associated with overall improvements in functional status, mobility, balance, and muscle performance in patients with rheumatoid arthritis. While clinical outcomes improved similarly across groups, the addition of custom-made insole mainly enhanced plantar pressure distribution, indicating a primary biomechanical benefit.

**Trial registration:**

The study was registered on the Clinical Trials Registry (registration number: NCT06753552).

**Table:** 

**Key Points** • *A six-week underwater ultrasound intervention resulted in significant improvements in functional status, mobility, balance, and plantar flexor muscle endurance in individuals with rheumatoid arthritis, regardless of treatment group.* • *The addition of custom-made foot insoles did not lead to superior clinical outcomes but provided distinct biomechanical advantages by significantly modifying Static Plantar Pressure Distribution.* • *Group-specific differences were primarily observed in plantar pressure parameters, indicating that insoles mainly influence biomechanical load redistribution rather than subjective clinical outcomes.* • *Underwater ultrasound may serve as a supportive therapeutic modality in rheumatoid arthritis, while the integration of custom-made foot insoles may offer additional biomechanical benefits, particularly in patients with foot involvement.*

## Introduction

Rheumatoid arthritis (RA) is a chronic autoimmune disease characterized by persistent synovial inflammation, progressive joint destruction, pain and deformities, and these pathological processes collectively lead to reduced mobility, functional impairment, and a marked deterioration in quality of life [1]. The foot and ankle joints, which are frequently affected in both the early and advanced stages of the disease, play a central role in the clinical course of RA, causing pain, instability, and deformity in a large proportion of patients [2]. Foot and ankle involvement is not limited to local symptoms but is also associated with altered gait patterns, increased energy expenditure, risk of falls, and significant limitations in activities of daily living [3]. In this context, effective management of foot-related symptoms is critical for maintaining functional independence and improving quality of life in individuals with RA [4].

Current RA treatment approaches primarily focus on systemic pharmacological treatments such as disease-modifying anti-rheumatic drugs and biological agents. While these treatments are effective in controlling inflammatory activity, they may not always sufficiently improve local musculoskeletal problems and functional limitations that arise in weight-bearing joints such as the feet and ankles. Therefore, local and adjunctive treatment methods are becoming increasingly important due to their complementary role to pharmacological treatment [5]. Among physical therapy and rehabilitation applications, therapeutic ultrasound stands out as a modality that supports analgesic and anti-inflammatory responses through its mechanical and thermal effects. Therapeutic ultrasound has been reported to reduce pain, increase soft tissue elasticity, and contribute to functional improvement in inflammatory joint diseases [6]. Underwater ultrasound, in particular, offers advantages in terms of ease of application and patient comfort in anatomically complex and sensitive areas such as the foot and ankle, thanks to more homogeneous contact and more effective transmission of ultrasound waves on irregular surfaces [7]. With these characteristics, underwater ultrasound is thought to be a potentially effective method in the management of RA-related foot symptoms.

In parallel, custom-made insoles are among the biomechanical interventions commonly used in the conservative treatment of RA-related foot problems. These orthotics aim to reduce stress in overloaded areas by optimizing plantar pressure distribution, improving foot alignment, and limiting compensatory mechanisms associated with deformities. The literature provides evidence that custom-made insoles reduce pain, improve gait parameters, and increase functional mobility [8]. However, these positive effects have mostly been reported at the biomechanical level, and the duration and degree of symptomatic relief may vary between individuals. Although underwater ultrasound therapy and custom insoles have individually demonstrated clinical benefits in RA, high-quality clinical evidence regarding the combined use of these two non-invasive approaches is limited [9, 10]. The current literature lacks randomized controlled trials evaluating the potential synergistic effects of combining these interventions on pain control, joint function, and mobility. However, simultaneously delivering local anti-inflammatory and analgesic benefits alongside biomechanical support may enable a more comprehensive management of RA-related foot symptoms.

The clarification of the effects of combining these two non-invasive interventions on clinical outcomes may contribute to the development of more comprehensive, targeted, and personalized rehabilitation strategies for individuals with RA. In this regard, the present randomized controlled trial aims to evaluate the effects of underwater ultrasound therapy alone and in combination with custom-made insoles on pain, functional capacity, and mobility in individuals with RA.

## Methods

This study was designed as a single-blind, randomized controlled trial with concealed allocation and evaluator blinding. The study was conducted in accordance with all CONSORT guidelines, and the necessary information has been reported accordingly. Potential participants were screened for eligibility, and the study was conducted at the School of Physiotherapy and Rehabilitation at Kırşehir Ahi Evran University. A blinded assessor evaluated all outcome measures before the intervention and immediately after the 6-week treatment period.

Eligible participants were randomly assigned to three parallel groups in a 1:1:1 ratio: a control group (CG) receiving no treatment, an underwater ultrasound group (UG) receiving underwater ultrasound, and an insole group (IG) receiving underwater ultrasound plus a custom-made insole. All interventions were administered by a physiotherapist with 10 years of clinical experience, who was aware of the group assignments. The outcome assessments were conducted throughout the study by a physiotherapist specializing in rheumatology with over 5 years of clinical experience, who was unaware of the participants’ allocation to groups. Ethical approval for the study was obtained from the Ethics Committee of Muş Alparslan University (Approval No: 197137), and the study was conducted in accordance with the principles outlined in the Declaration of Helsinki. Written and verbal informed consent was obtained from all participants prior to their enrollment in the study.

## Participants

The study included individuals aged 18 years and older who met the American College of Rheumatology diagnostic criteria, had mild-to-moderate RA activity (DAS-28 score > 3.2 and < 5.1), and experienced foot pain. Additionally, participants were required to have received no physical therapy or rehabilitation treatment in the month prior to inclusion in the study. Individuals with any of the following characteristics were excluded from the study: degenerative joint diseases or structural/anatomical abnormalities in the feet, acute flare-up period, use of walking aids, neurological diseases, history of malignancy, cognitive impairment, pregnancy, previous foot surgery, other autoimmune diseases involving foot ulcers, and conditions contraindicating ultrasound therapy (infection, fever, osteomyelitis, etc.).

## Interventions

All interventions were performed by physiotherapists specializing in rheumatological rehabilitation, and the treatment protocols were identical for all participants. To ensure intervention standardization and treatment fidelity, all physiotherapists received prior training on the study protocols, including detailed instructions on ultrasound application parameters and insole use. A standardized treatment protocol was strictly followed, with predefined parameters such as ultrasound intensity, duration, application areas, and patient positioning. Participation records and self-reports obtained from participants were used to monitor adherence to the interventions. Participants were considered compliant if they attended at least 80% of the planned underwater ultrasound sessions and reported consistent use of the orthosis during daily activities. Periodic supervision and protocol checks were conducted by the research team to ensure consistency across sessions and minimize inter-therapist variability. To handle dropouts and missing data, a per-protocol analysis was utilized; therefore, only data from participants who fully completed the study and met these compliance criteria were included in the final analysis. The study was conducted with three groups, where the control group (CG) was selected from a waiting list and received no additional physiotherapy interventions during the study period.

## Underwater ultrasound treatment

For 6 weeks, participants in both the UG and IG groups received 20 sessions of pulsed underwater ultrasound therapy. The treatment was administered using a Chattanooga® ultrasound device in tap water maintained at 35–36 °C. The ultrasound probe was positioned approximately 2 cm away from the treated surface. The ultrasound was applied in pulsed mode at a spatial–temporal average intensity of 0.9 W/cm^2^, using a treatment probe size of 4.2 cm^2^, to reduce excessive thermal load while enhancing mechanical and anti-inflammatory effects. The probe was moved in small circles over the ankle joint, first metatarsal region, and medial longitudinal arch of the painful foot. The treatment session was administered 5 times per week for 7 min [11].

## Custom-made insole treatment

In addition to in-water ultrasound, a custom-made insole was used in the IG, which was specifically designed based on static plantar pressure analyses. The insoles were manufactured using CNC technology from semi-rigid thermoplastic material and featured medial longitudinal arch support, heel cup, and elements that redistribute foot pressure [12]. Throughout the intervention period, participants were instructed to use their insoles during any weight-bearing activities, such as standing and walking.

## Outcome measurements

### Functional status

The Health Assessment Questionnaire (HAQ) is a patient-reported measure consisting of 20 questions that assesses individuals’ functional status and activities of daily living, particularly in rheumatological diseases [13]. The questionnaire covers eight core functional domains and is scored by assigning each item a score between 0 (no difficulty) and 3 (unable to perform). The total HAQ score ranges from 0 to 3, with higher scores indicating greater functional limitations [13, 14].

#### Static plantar pressure distribution assessment

Static Plantar Pressure Distribution (SPPD) was assessed using a pedobarography device. During measurements, participants were positioned on the measurement platform with both feet in a natural and comfortable standing posture, facing forward. Within the scope of static measurements, the plantar surface was analyzed by dividing it into the right-front (RF), right-back (RB), left-front (LF), and left-back (LB) regions [15]. The data obtained was recorded as the percentage of pressure distribution falling on each plantar region.

### Assessment of foot function and symptom severity

The Manchester–Oxford Foot Questionnaire (MOXFQ) is a 16-item patient-reported measure developed to assess foot and ankle-specific problems [16]. The questionnaire comprises three subscales: pain, walking/standing, and social interaction. Each item is scored from 0 to 4; subscale and total scores are converted to a 0–100 scale. Higher scores indicate poorer foot function and greater symptom severity.

### Balance assessment

Individuals’ balance performance was assessed using the Berg Balance Scale (BBS). The test consists of 14 functional tasks, each item being scored between 0 and 4 points. The total score ranges from 0 to 56, with higher scores indicating better balance performance and lower scores indicating impaired balance and increased risk of falling [17].

### Mobility

Individuals’ functional mobility and risk of falling were assessed using the Timed Up and Go Test (TUG). In the test, the individual is asked to stand up from a chair, walk 3 m, turn around, and sit back down on the chair. The test time is recorded in seconds; longer times indicate reduced functional mobility and increased risk of falling [18].

### Muscle strength and endurance

The Single Heel-Rise Test (SHRT) was used to assess plantar flexor muscle performance. Participants stood barefoot on a flat, firm surface and were instructed to maintain balance while standing on one leg. During the test, participants were asked to rise onto the forefoot by lifting the heel as high as possible. Each limb was assessed separately. The maximum vertical height of heel elevation from the floor was measured using a tape measure and recorded in centimeters (cm). The test was terminated if the participant experienced loss of balance or pain. All measurements were performed by the same examiner to ensure standardization [19].

#### Assessment of ankle edema

To assess edema around the ankle circumference (AC), the 8-shaped circumference measurement method was used with a tape measure. In this method, the tape measure is placed in an 8-shape by passing it through the anatomical reference points of the ankle, and the measurement value obtained quantitatively reflects the level of edema in the AC [11].

### Sample size

The sample size calculation was performed using GPower* software (version 2.2; Universität Düsseldorf, Germany). The analysis was conducted based on an 80% statistical power (1 − *β* = 0.80), a 5% significance level (*α* = 0.05), and Cohen’s *d* = 0.80, which is considered a large effect size [6]. As a result of this calculation, it was determined that at least 25 participants in each group, totalling 75 participants, were required for the study to detect a large effect.

### Randomization and blinding

This study has a single-blind, randomized controlled design. Participants who met the inclusion criteria and provided written informed consent were randomized in a 1:1:1 ratio to three groups: CG, UG, and IG.

The randomization process was conducted by an independent researcher, using a computer-based random number generation method, independent of the treatment and evaluation processes. The block randomization method was applied to maintain numerical balance between groups. To ensure allocation concealment, group assignments were sequentially numbered, stored in opaque, sealed envelopes, and opened for each participant only after the completion of the evaluation process. The study employed a single-blind design, meaning that the assessors conducting the outcome measurements were unaware of which group the participants had been assigned to. Due to the nature of the interventions, it was not possible to blind the participants or the implementing therapists. However, all assessments were conducted by a blinded assessor in accordance with standardized measurement protocols, with the aim of minimizing measurement bias.

### Statistical analysis

Statistical analyses were performed using SPSS software (version 25.0). The normality of data distribution was evaluated using both visual (histograms and probability plots) and analytical methods (Shapiro–Wilk test). Descriptive statistics for normally distributed continuous variables were expressed as mean and standard deviation (SD). A one-way analysis of variance (ANOVA) was utilized to compare the baseline measured values among the groups (CG, UG, and IG). To evaluate changes over time and group-by-time interactions across the groups, a two-way mixed-design ANOVA with repeated measures was conducted. Eta squared (*η*^2^) was calculated to classify effect sizes as 0.02 (small), 0.13 (moderate), and 0.26 (large). Effect sizes were interpreted only when a statistically significant difference was detected [20]. The level of statistical significance was set at *p* < 0.05.

## Results

**Fig. 1:**
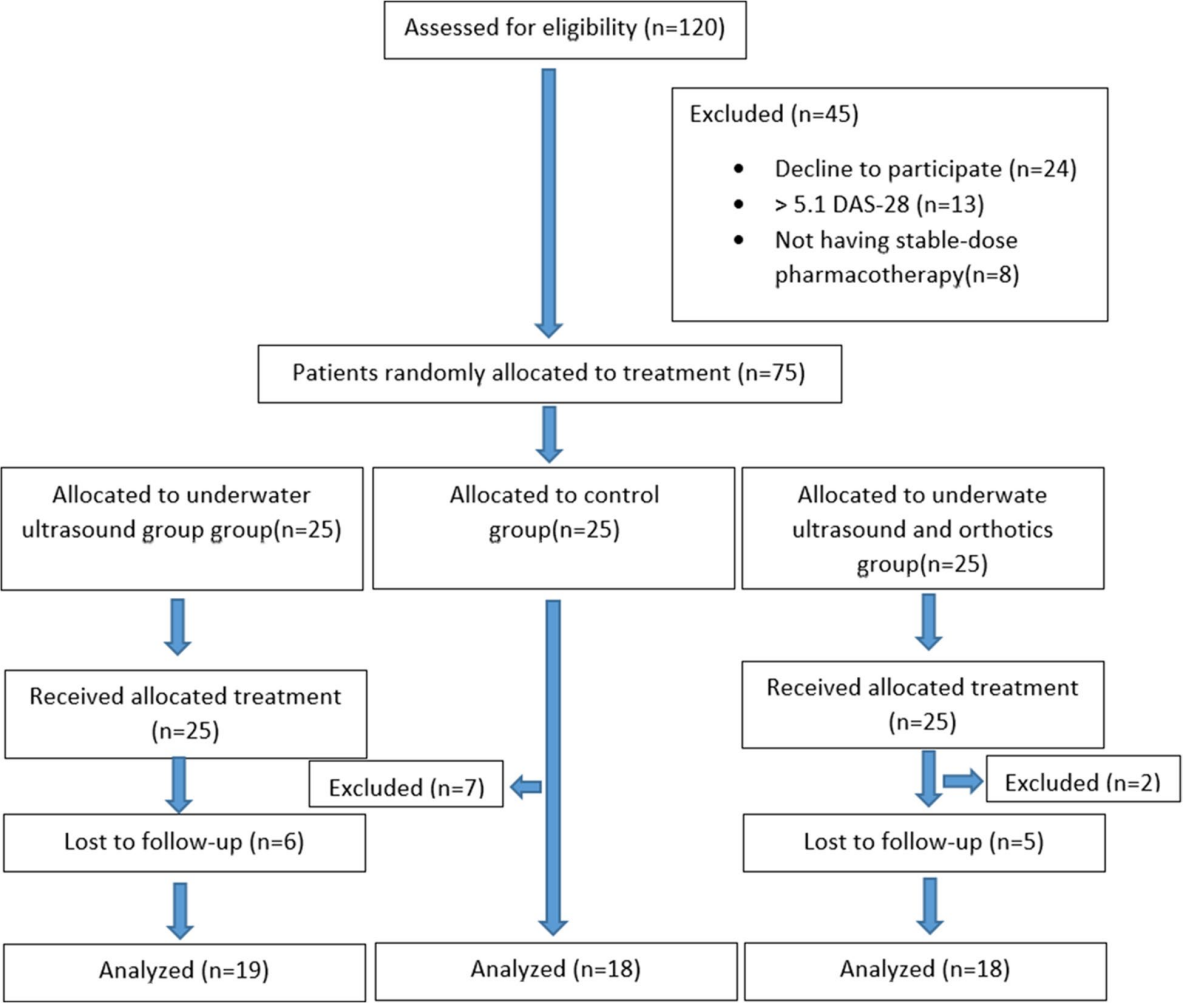
Flow chart of the study

Patients who met the inclusion criteria were randomized using the closed-envelope method and assigned to one of three groups (Fig. 1). A total of 120 patients were assessed for eligibility. Of these, 45 patients were excluded due to declining participation (*n* = 24), having a DAS-28 score > 5.1 (*n* = 13), or not receiving stable-dose pharmacotherapy (*n* = 8). Seventy-five patients were randomly allocated into three groups: ultrasound group (UG, *n* = 25), control group (CG, *n* = 25), and intervention group (IG, *n* = 25). All allocated participants initially received the assigned interventions. During the follow-up period, participants were excluded due to loss to follow-up (UG, *n* = 6; CG, *n* = 7; IG, *n* = 5). To handle dropouts and missing data, a per-protocol analysis was utilized; therefore, any participants with incomplete data were completely excluded, and only data from 19 patients in the UG, 18 in the CG, and 18 in the IG were included in the final analysis.

Comparisons of the demographic and clinical characteristics of the groups are presented in Table 1. No statistically significant differences were observed between the groups in terms of age, height, weight, body mass index (BMI), or duration of illness (*p* > 0.05). These findings indicate that the groups are comparable in terms of their baseline demographic and clinical characteristics.

**Table 1:** Comparison of demographic and clinical characteristics between groups

Variable	CG (*n* = 18) Mean ± SD	UG (*n* = 19) Mean ± SD	IG (*n* = 18) Mean ± SD	*F*	*p*
Age (years)	48 ± 9.02	45 ± 9.15	49 ± 12.17	0.608	0.548
Height (m)	1.63 ± 0.09	1.63 ± 0.10	1.63 ± 0.07	0.005	0.995
Weight (kg)	80.98 ± 14.07	80.42 ± 12.66	83.96 ± 11.92	0.395	0.675
BMI (kg/m^2^)	31.91 ± 4.80	33.45 ± 5.01	33.41 ± 5.80	0.512	0.602
Duration of disease (years)	10 ± 8.19	9 ± 6.24	12 ± 6.55	0.837	0.439

*CG* control group, *UG* ultrasound group, *IG* ultrasound + insole group, *BMI* body mass index, *SD* standard deviation, *F* statistic derived from one-way ANOVA

The comparisons of the measurement data of the groups in terms of time, group, and group-by-time interactions are presented in Table 2. To mitigate the risk of Type I error due to multiple comparisons, a Bonferroni correction was applied to the analyses. Accordingly, when the changes in the measurement data over time were examined in the CG, UG, and IG, a statistically significant time effect was detected in all parameters except for the AC (right and left) variables (*p* < 0.05). In the comparisons made in terms of group-by-time interaction, statistically significant differences were determined between the groups for the HAQ and SPPD (RF, RB, and LB) parameters (*p* < 0.05), while no significant interaction was observed in the other variables (*p* > 0.05).

**Table 2:** Comparison of pre- and post-treatment outcomes among groups

Outcome Measure	Time	CGᵃ (*n* = 18) Mean ± SD	UGᵇ (*n* = 19) Mean ± SD	US + IGᶜ (*n* = 18) Mean ± SD	Time (*p*)	Group × Time (*p*/*F*)	Post-hoc	*η*^2^
**HAQ**	Before	1.20 ± 0.31	1.24 ± 0.43	1.22 ± 0.40	< 0.001	0.002/6.87	–	0.209
	After	0.89 ± 0.40	0.87 ± 0.38	0.62 ± 0.38				
**MOFQ**	Before	36 ± 7.71	34 ± 10.80	41 ± 9.08	< 0.001	0.336/1.11	–	0.041
	After	25 ± 6.62	25 ± 11.28	27 ± 11.18				
**TUG (s)**	Before	8.94 ± 0.80	8.97 ± 0.96	9.30 ± 1.15	< 0.001	0.723/0.32	–	0.012
	After	7.75 ± 0.63	7.90 ± 0.61	7.91 ± 0.62				
**BBS**	Before	50 ± 3.08	50 ± 3.78	49 ± 3.21	< 0.001	0.401/0.93	–	0.035
	After	53 ± 2.30	53 ± 3.18	53 ± 3.63				
**SPPD (RF-%)**	Before	21.21 ± 2.07	21.86 ± 1.23	21.48 ± 1.84	< 0.001	< 0.001/39.10	a–b, a–c	0.601
	After	21.13 ± 2.10	23.92 ± 1.41	24.81 ± 1.42				
**SPPD (RB-%)**	Before	29.11 ± 2.73	30.10 ± 1.14	30.74 ± 1.62	< 0.001	< 0.001/29.65	–	0.533
	After	29.19 ± 2.49	28.07 ± 1.18	27.53 ± 2.26				
**SPPD (LF-%)**	Before	19.69 ± 3.30	22.42 ± 1.61	22.23 ± 4.29	< 0.001	0.074/2.73	a–b, a–c	0.095
	After	19.95 ± 3.31	24.12 ± 1.56	23.67 ± 2.45				
**SPPD (LB-%)**	Before	29.99 ± 2.18	25.63 ± 1.60	25.60 ± 3.18	< 0.001	0.021/4.18	a–b, a–c	0.139
	After	29.74 ± 2.14	23.88 ± 1.93	23.99 ± 1.91				
**SHRT (R-cm)**	Before	7.0 ± 1.38	7.0 ± 0.78	6.0 ± 1.73	< 0.001	0.959/0.04	b–c	0.002
	After	8.0 ± 1.46	8.0 ± 1.26	7.0 ± 1.58				
**SHRT (L-cm)**	Before	7.19 ± 1.49	7.21 ± 1.51	6.14 ± 2.80	< 0.001	0.769/0.26	–	0.010
	After	8.83 ± 1.38	9.03 ± 0.98	8.17 ± 1.95				
**AC (R-cm)**	Before	54.56 ± 4.94	54.05 ± 3.69	52.92 ± 4.15	0.469	0.833/0.18	–	0.007
	After	54.0 ± 5.25	54.0 ± 3.52	52.0 ± 3.37				
**AC (L-cm)**	Before	53.0 ± 5.45	53.0 ± 3.43	52.0 ± 4.66	0.441	0.762/0.27	–	0.010
	After	53.0 ± 5.27	52.0 ± 3.36	51.0 ± 3.79				

*CG* control group, *UG* ultrasound group, *IG* ultrasound + insole group, *SD* standard deviation, *HAQ* Health Assessment Questionnaire, *MOFQ* Manchester–Oxford Foot Questionnaire, *TUG* Timed Up and Go Test, *BBS* Berg Balance Scale, *SPPD* Static Plantar Pressure Distribution, *RF* right-front, *LF* left-front, *RB* right-back, *LB* left-back, *SHRT* Single Heel-Rise Test, *AC* ankle circumference, *η*^2^ partial eta squared

In the change difference comparisons for parameters with significant interactions, the highest improvements in the HAQ and SPPD (RF and RB) variables were observed in the IG, while the highest change in the SPPD (LB) variable was observed in the UG (Table 2). When analyzing these parameters in terms of effect sizes, a moderate effect size was observed for the HAQ parameter, whereas a large effect size was observed for the SPPD variables. Considering the amount of change in the HAQ scores and the minimal clinically ımportant difference (MCID), the treatments demonstrated a substantial and clinically meaningful impact on the functional status of the patients. Furthermore, the large effect sizes observed in SPPD signify robust biomechanical adaptations that are highly relevant to clinical recovery.

## Discussion

This study, which examines the effects of underwater ultrasound and insole use in individuals with RA experiencing foot pain, is, to our knowledge, the first randomized controlled trial in this field. Our findings indicate that a 6-week underwater ultrasound application is associated with significant improvements in functional status, mobility, and balance, independent of the treatment group. In contrast, the addition of custom-made insoles primarily provided biomechanical advantages in SPPD. While all groups showed time-dependent improvements in clinical outcomes such as functional impairment, mobility, and balance, differences between the groups were particularly evident in SPPD. In the UG and the IG, forefoot pressure increased while rearfoot pressure decreased, with this effect being more pronounced in the IG. These results suggest that underwater ultrasound contributes to overall clinical improvement, while insole integration synergistically supports functional gains by regulating mechanical load distribution.

The number of studies examining the use of underwater ultrasound in individuals with RA is limited, and the majority of existing studies have focused on the hand and wrist joints [6, 21, 22]. These studies report that underwater ultrasound application reduces pain and inflammation markers, particularly CRP levels, in the short term; however, it has positive but limited effects on hand function and grip strength. Therefore, it is emphasized that ultrasound therapy should be considered primarily as an adjunctive modality in RA. Nevertheless, the foot is one of the joints frequently affected in individuals with RA and is of great clinical importance due to its direct impact on walking, balance, and overall mobility [23, 24].

In our study, the absence of significant differences between the groups in terms of age, BMI, and disease duration suggests that the findings may be largely related to the interventions applied. Although functional improvement was observed in all groups, the most pronounced improvements were achieved in the group that used insoles in addition to underwater ultrasound. This finding indicates that the biological effects of underwater ultrasound in reducing pain and inflammation, when combined with the biomechanical support provided by insoles, may enhance functional improvement. In contrast, foot-related pain and functional limitations decreased similarly across all groups, and no significant group × time interaction was detected for these variables. This suggests that pharmacological treatment and time-dependent improvements play a decisive role in managing foot symptoms in individuals with RA, and that the isolated additional contribution of ultrasound therapy to these specific parameters may be limited. Similarly, a meta-analysis including patients with plantar fasciitis reported that therapeutic ultrasound, either alone or in combination with exercise, did not provide a significant advantage in terms of pain reduction [23]. Furthermore, a randomized controlled trial demonstrated that active ultrasound administered in conjunction with stretching exercises did not provide any additional benefit in terms of pain and functional outcomes compared to sham ultrasound [25].

Regarding functional mobility and balance, parameters improved significantly in all groups; however, no superior difference was detected among them. A previous study involving individuals with RA reported that both individual and group-based exercise programs provided significant improvements in pain, balance, and mobility scores, but neither type of exercise showed a clear superiority over the other [26]. Although pain, balance, and functional outcomes improved similarly across groups, SPPD demonstrated a significant group × time interaction, particularly in the UG and IG. This finding suggests that the use of insoles has a more pronounced effect on the redistribution of biomechanical load rather than solely on subjective clinical outcomes. The observed increase in forefoot pressure and decrease in rearfoot pressure can be explained by the combination of ultrasound’s biological effects, which increase soft tissue elasticity, and the mechanical effects of the insole, which aims to redistribute plantar pressure. This demonstrates that the biological effects of ultrasound and the mechanical properties of the insole work synergistically.

The synergistic effect of this combined therapy can be explained through a dual-pathway mechanism. Biologically, underwater ultrasound exerts its effects via non-thermal acoustic streaming and cavitation, which increase cell membrane permeability and stimulate fibroblast activity, thereby enhancing the elasticity of periarticular soft tissues. This “softening” effect on the connective tissue likely creates a more receptive environment for biomechanical realignment [27]. Biomechanically, the custom-made insoles leverage this increased tissue flexibility to more effectively redistribute vertical ground reaction forces away from the inflamed rearfoot toward the forefoot [28]. By reducing the cumulative mechanical load on sensitized synovial structures, the insole minimizes the ongoing micro-trauma that typically exacerbates RA-related inflammation, while the ultrasound simultaneously accelerates the local healing response. Thus, the combined approach addresses both the symptomatic inflammatory triggers and the underlying mechanical stressors that perpetuate foot dysfunction in RA.

From a functional perspective, this biomechanical optimization provides a more stable base of support. This stability is a critical precursor for performing daily mobility tasks safely, potentially explaining why patients in the IG showed robust improvements in balance and mobility tests [29]. While underwater ultrasound addresses the inflammatory and symptomatic aspects, the insoles provide the necessary structural framework to maintain functional gains.

Despite these targeted biomechanical and functional advantages observed in the intervention groups, it is important to address the improvements also noted in the CG. The improvements observed in the CG can be explained by several factors inherent to the clinical course of RA and the study design. First, all participants continued to receive standard pharmacological management throughout the study period, which is known to reduce inflammation and pain, thereby contributing to functional gains over time. Second, RA symptoms often exhibit natural fluctuations, and regression toward the mean may partially account for improvements independent of the intervention. Third, repeated exposure to functional tests may have led to a learning or familiarization effect, resulting in better performance in mobility and balance assessments.

Previous studies on RA and plantar pressure distribution have reported that insoles can alter plantar loading patterns and that therapeutic ultrasound can facilitate biomechanical adaptations by affecting the mechanical properties of soft tissue [30, 31]. Conversely, a randomized controlled trial has shown that insoles do not significantly alter plantar pressure distribution, despite improving pain and walking performance [32]. This highlights that biomechanical changes may not always parallel subjective clinical gains. Furthermore, it has been reported that structural foot deformities seen in RA are associated with altered plantar loading patterns [33, 34], supporting the notion that biomechanical factors play an important role in shaping the efficacy of interventions.

The improvement in the Single Heel-Rise Test (SHRT) observed in our study is consistent with existing evidence showing that increased plantar flexor muscle endurance is positively associated with functional walking capacity and ankle power output [35, 36]. SHRT is recognized as a reliable and valid clinical measurement tool for assessing plantar flexor muscle performance [37]. Our study did not detect any significant changes in ankle circumference (AC) measurements over time or between groups. This finding may be attributed to the low baseline levels of clinically evident inflammatory edema in the included patients and the limited sensitivity of circumferential measurements in detecting subclinical inflammatory changes. Another possible factor is that the short-term anti-edema effects of therapeutic ultrasound may be limited in chronic RA [35]. Previous studies have shown that inflammatory activity in the ankle and foot joints of individuals with RA does not always correlate strongly with clinical findings such as swelling or circumferential increase [38, 39]. This may explain why no significant change was observed in AC measurements, despite improvements in other clinical and functional parameters.

This study has several limitations. First, the follow-up period was limited to 6 weeks, which restricts conclusions regarding the long-term effectiveness of the interventions. This is particularly important, as both ultrasound therapy and orthotic interventions may demonstrate delayed or sustained effects over time. Second, the single-blind design represents an important limitation, as neither participants nor treating therapists were blinded to group allocation. This may have introduced performance and placebo biases; the novelty of receiving specialized custom-made insoles and a technology-based intervention like underwater ultrasound could have influenced participants’ expectations. These psychological factors may have particularly affected patient-reported outcomes such as HAQ and MOXFQ scores, where perceived benefits often influence responses. Although assessor blinding was implemented to minimize detection bias, the potential impact of performance bias on functional tests (e.g., TUG and BBS) should be considered. Third, to minimize confounding effects from comorbidities, we strictly excluded individuals with severe conditions; however, detailed information on specific concomitant medications was not analyzed in the final dataset, which may have acted as an unmeasured confounder. Fourth, the discrepancy between the initially planned sample size and the final number of participants due to attrition may have reduced the statistical power, increasing the risk of Type II error. Additionally, the absence of a unidimensional pain assessment tool (e.g., VAS or NRS) may limit the sensitivity of pain-specific interpretations. Finally, the inclusion of only individuals with mild-to-moderate disease activity (DAS-28 > 3.2 and < 5.1) limits the generalizability of the results to the broader RA population, especially those with severe joint involvement.

## Conclusion

This randomized controlled trial demonstrates that a 6-week underwater ultrasound intervention improves functional status, mobility, balance, and muscle endurance in individuals with RA. Although clinical outcomes improved similarly across all groups, the addition of a custom-made insole provided significant biomechanical benefits by optimizing SPPD. These findings indicate that while underwater ultrasound supports overall clinical improvement, the insole primarily contributes to the redistribution of biomechanical load, thereby establishing a structural foundation that minimizes joint stress and enhances the overall functional efficacy of the rehabilitation process.

## Declarations

### Study setting

The study WAS conducted at Kırşehir Ahi Evran University, School of Physical Therapy and Rehabilitation, in collaboration with the Rheumatology Outpatient Clinic of Kırşehir Training and Research Hospital.

### Study design

Single-blind, randomized controlled trial.

### Trial registration

Registered at ClinicalTrials.gov Identifier: NCT06168760.
